# Characterization of Host Protein Prohibitin‐2 Interacting With *Cryptosporidium parvum* Cyclophilin 23

**DOI:** 10.1155/tbed/8933271

**Published:** 2026-08-12

**Authors:** Mengfei Xu, Qi Feng, Xichen Zhang, Qi Zhao, Qile Yu, Jianhua Li, Pengtao Gong, Xiaocen Wang, Xin Li, Xu Zhang, Nan Zhang

**Affiliations:** ^1^ State Key Laboratory for Diagnosis and Treatment of Severe Zoonotic Infectious Diseases, Key Laboratory for Zoonosis Research of the Ministry of Education, Institute of Zoonosis, and College of Veterinary Medicine, Jilin University, Changchun 130062, China, jlu.edu.cn

## Abstract

*Cryptosporidium parvum* is a prevalent protozoan pathogen responsible for moderate to severe diarrhea in humans and animals, posing a significant threat to global public health. Host–parasite interactions play a crucial role in the infection process of *C. parvum*. Cyclophilin 23 (CpCyP23), a secreted protein of *C. parvum*, is implicated in parasite development and pathogenicity. As a secretory protein, its function often relies on interactions with host cell proteins. However, it remains unclear whether CpCyP23 has host interaction partners and what specific roles such interactions play during infection. In this study, using CpCyP23 as bait, we screened for potential interacting proteins in HCT‐8 cells through pull‐down assays combined with mass spectrometry (MS) analysis. The interaction was subsequently confirmed by co‐immunoprecipitation (Co‐IP) and bimolecular fluorescence complementation (BiFC) assays. Immunofluorescence (IF) was employed to determine subcellular localization. Furthermore, after confirming the altered expression of the interacting protein during parasite infection, we analyzed its impact on parasite infection using small interfering RNA (siRNA) knockdown and overexpression techniques, coupled with parasite infection assays. The results identified the host cell protein Prohibitin‐2 (PHB2) as an interacting partner of CpCyP23. Co‐IP and BiFC assays validated their interaction, and IF revealed their colocalization in the cytoplasm of HCT‐8 cells. The mRNA level of PHB2 was significantly upregulated at 3 and 12 h postinfection (hpi) with *C. parvum*. Functional experiments further demonstrated that the expression level of PHB2 positively correlates with the infection burden of *C. parvum*. In summary, this study reveals an interaction between the host cell protein PHB2 and CpCyP23 and identifies PHB2 expression level as a key host factor significantly influencing *C. parvum* infection. These findings provide experimental evidence and a potential target for elucidating the pathogenic mechanisms of *C. parvum* and developing novel anti‐infection strategies.

## 1. Introduction

*Cryptosporidium parvum* is a significant apicomplexan parasitic protozoan that causes moderate to severe diarrhea, known as cryptosporidiosis, in both humans and animals. This pathogen poses a major threat to global public health, with particularly severe impacts on infants, immunocompromised individuals, and populations in developing countries [1]. The control of this disease faces substantial challenges due to the current lack of effective therapeutic drugs and vaccines. Nitazoxanide offers only partial efficacy in immunocompetent patients and fails to achieve complete parasite clearance [2]. *C. parvum* targets host intestinal epithelial cells and exhibits a unique intracellular yet extracytoplasmic parasitic niche [3]. However, the molecular mechanisms underlying its invasion of host cells remain incompletely understood.

Protein interactions between pathogens and their hosts are the primary factors mediating infection. Pathogens often secrete specific virulence factors to “hijack” key host cell proteins, thereby subverting host defense mechanisms and promoting their own invasion and survival [4, 5]. Several *C. parvum* proteins involved in host interactions have been identified, such as elongation factor‐1α, P23, and gp40 [3, 6, 7]. Cyclophilins constitute a highly evolutionarily conserved protein family possessing peptidyl‐prolyl cis‐trans isomerase activity. They are involved not only in proper protein folding but also play crucial roles in the infection and development of various apicomplexan parasites, including *Trypanosoma cruzi*, *Leishmania donovani*, *Giardia intestinalis*, and *Toxoplasma gondii* [8–11]. Studies indicate that the *C. parvum* cyclophilin family comprises seven members. Among them, Cyclophilin 23 (CpCyP23) is a secreted protein present in extracellular vesicles released by sporozoites and plays an important role during infection [10, 12]. Research suggests that sporozoite surface proteins interact directly with host cells, while secreted proteins are released or delivered into host cells, enabling interactions with host membrane or cytoplasmic proteins [13, 14]. It is therefore plausible that CpCyP23 may interact with host cell proteins.

In this study, using purified CpCyP23 as bait, we performed a glutathione S‐transferase (GST) pull‐down method to identify candidate host cell proteins interacting with CpCyP23. Subsequent bioinformatics analysis identified Prohibitin‐2 (PHB2) as a potential host interaction partner. The interaction between CpCyP23 and PHB2 was confirmed by bimolecular fluorescence complementation (BiFC) and co‐immunoprecipitation (Co‐IP) assays. Further functional investigations revealed that the host protein PHB2 participates in the parasite infection process and is essential for the parasite’s endogenous development.

## 2. Materials and Methods

### 2.1. Parasites, Cells, and Experimental Animals

The *C. parvum* isolate (GP60 genotype IIaA15G2R1) used in this study was maintained and propagated in our laboratory. Human ileocecal adenocarcinoma cells (HCT‐8) and human embryonic kidney 293T (HEK293T) cells were purchased from the Chinese Academy of Sciences.

### 2.2. Preparation of HCT‐8 Cell Total Protein

Total protein was extracted from HCT‐8 cells grown in culture dishes. Following medium removal, the cell monolayer was washed three times with ice‐cold phosphate‐buffered saline (PBS). An appropriate volume of RIPA lysis buffer (Beyotime, Shanghai, China) supplemented with 1 mM phenylmethylsulfonyl fluoride (PMSF) was then added to the dish to cover the cells. After incubation on ice for 30 min with periodic agitation, the lysate was scraped into a microtube and clarified by centrifugation at 12,000 × *g* for 10 min at 4°C. The supernatant (total protein extract) was collected and stored at −80°C.

### 2.3. Identification of Potential CpCyP23‐Interacting Proteins

To identify interacting partners of CpCyP23, a GST pull‐down assay was performed. Briefly, the recombinant GST‐CpCyP23 fusion protein was immobilized by incubation with glutathione magnetic beads (Yeasen, Shanghai, China) for 2 h at room temperature. After washing to remove the unbound bait protein, the beads were incubated with total protein from HCT‐8 cells for an additional 2 h to allow protein–protein interactions. The bead‐bound complexes were then isolated using a magnetic stand. To eliminate nonspecifically adsorbed proteins, three washes with the wash buffer were performed. The bead‐bound complexes were resuspended in 1 × protein loading buffer and boiled for 10 min. The eluted proteins were then collected as the CpCyP23 pull‐down product by retaining the supernatant on a magnetic stand. The eluate was resolved by SDS‐PAGE, and the gel was subjected to silver staining using a commercial kit (Yeasen). The gel bands from the experimental group were excised and subsequently characterized by mass spectrometry (MS) at BGI Tech (Shenzhen, China). A parallel assay using the purified GST protein as the bait served as a negative control to account for background binding.

### 2.4. Selection of Host Protein for Validation

To prioritize host proteins identified by MS for validation, a multistep strategy was applied. First, high‐confidence protein identifications were prioritized based on a set of stringent criteria, including relative abundance, sequence coverage, exp. *q*‐value (false discovery rate, FDR, denoted as *q*‐value < 0.01) and the number of unique peptides (detailed MS parameters are provided in Supporting Information 1: Table S1). Second, we prioritized host proteins with predicted cytoplasmic localization as the most plausible interaction partners, given that CpCyP23 contains a secretory signal peptide and is detected in *C. parvum* sporozoite extracellular vesicles, and apicomplexan parasites are well‐established to secrete effector proteins into host cells to modulate their functions [15–17]. Third, functional enrichment analysis was performed on the remaining proteins, and those associated with pathways known to be modulated by *C. parvum* during host cell infection, such as the apoptosis pathway [18], were selectively retained. PHB2 met all three selection criteria and was prioritized as a validation candidate due to its role in pathways such as apoptosis and cell cycle regulation [19].

### 2.5. Plasmid Construction

To substantiate the physical association between CpCyP23 and human PHB2—a candidate identified via proteomic screening—we performed Co‐IP and BiFC assays. The coding sequences for CpCyP23 and PHB2 were amplified by PCR using pGEX‐4T‐CpCyP23 and HCT‐8 cDNA as templates, respectively. For the Co‐IP experiments, these genetic sequences were integrated into pcDNA3.1‐HA and pcDNA3.1‐FLAG backbones to produce the fusion constructs pcDNA3.1‐HA‐CpCyP23 and pcDNA3.1‐FLAG‐PHB2. Simultaneously, these coding sequences were ligated into pBiFC‐VC155 and pBiFC‐VN173 vectors for BiFC. Furthermore, unmodified pcDNA3.1 vectors harboring CpCyP23 or PHB2 were synthesized for subsequent overexpression and subcellular distribution analyses. The oligonucleotide sequences synthesized for these cloning procedures are documented in Supporting Information 2: Table S2.

### 2.6. BiFC

The interaction between CpCyP23 and PHB2 was assessed using the BiFC system. For this assay, HEK293T cells were cultured in 6‐well plates to ~70%–80% confluence. The cells were then cotransfected, using Lipofectamine 2000 reagent, with a 1:1 mixture of the pBiFC‐VN173‐CpCyP23 and pBiFC‐VC155‐PHB2 constructs. Twenty‐4 h posttransfection, cells were processed for fixation. The culture medium was removed, and the cell monolayer was sequentially washed with PBS, fixed with 4% paraformaldehyde for 10 min at room temperature, and then washed again with PBS. Cell nuclei were counterstained by applying an antifade mounting medium containing DAPI (Beyotime). Fluorescent signals resulting from reconstituted Venus fluorescence were visualized and captured using an Olympus IX83 P2ZF super‐resolution microscope (Olympus, Tokyo, Japan).

### 2.7. Co‐IP

To confirm the interaction between CpCyP23 and PHB2, a Co‐IP assay was conducted in HEK293T cells. Cells were cultured in 6‐well plates until they attained 70%–80% confluence for subsequent transfection. Transient cotransfection with plasmids pcDNA3.1‐HA‐CpCyP23 and pcDNA3.1‐FLAG‐PHB2 (1:1 ratio) was performed using Lipofectamine 2000 (Thermo Scientific, Waltham, MA, USA). After 48 h, the growth medium was aspirated, and the cell monolayers were harvested into 100 μL of Western/IP lysis buffer (Beyotime) fortified with 1 mM PMSF. After incubation on ice for 30 min, the lysates were cleared by centrifugation (12,000 × g, 10 min, 4°C). The resulting supernatants were then engaged with anti‐FLAG or anti‐HA magnetic beads (MCE, Monmouth Junction, NJ, USA) for a 2‐h association period at ambient temperature. Postinteraction, the beads underwent three sequential TBST rinses before the immunocomplexes were eluted by thermal denaturation in a 1 × protein loading buffer. Finally, the eluates were then analyzed by Western blotting.

### 2.8. Western Blotting

Following electrophoretic fractionation via SDS‐PAGE, target proteins were electroblotted onto PVDF membranes employing a wet‐transfer apparatus. To prevent nonspecific interactions, the membranes were submerged in a blocking buffer containing 5% (w/v) BSA for a 2‐h period at ambient temperature. Subsequently, the membrane was incubated overnight at 4°C with anti‐FLAG (1:1000, Servicebio, GB12938‐100, Wuhan, China), anti‐HA (1:4000, Proteintech, 66006‐2‐Ig, Wuhan, China) (1:2000, Proteintech, 51064‐2‐AP), anti‐His antibody (1:2000, Proteintech, 66005‐1‐Ig), and anti‐GAPDH antibody (1:10000, Proteintech, 60004‐1‐Ig). After three successive rinses with TBST to remove residual antibodies, the membranes were labeled with HRP‐linked goat anti‐rabbit (1:5000, Proteintech, SA00001‐2) or anti‐mouse IgG (1:5000, Proteintech, SA00001‐1) for 1 h at room temperature. The resulting immunoreactive complexes were developed using an ECL chemiluminescence reagent (Yeasen) and captured via the Clinx ChemiScope 6300 imaging platform (Clinx, Shanghai, China).

### 2.9. Expression of His‐PHB2 and Polyclonal Antibody Generation

The recombinant His‐tagged PHB2 protein was expressed using the pET32a vector in *Escherichia coli* BL21(DE3) cells. Protein induction was carried out under standard conditions (1 mM IPTG, 37°C, 6 h), followed by affinity purification using a Ni‐TED agarose purification resin (Sangon Biotech, Shanghai, China). For polyclonal antibody production, Japanese White rabbits were subcutaneously immunized with an initial dose of 400 µg of His‐PHB2 protein emulsified in Freund’s complete adjuvant. This was followed by two booster injections with the antigen emulsified in Freund’s incomplete adjuvant on days 14 and 28. Serum was collected on day 35 postimmunization, with preimmune serum serving as the control.

### 2.10. Immunofluorescence (IF) Analysis

The subcellular colocalization of PHB2 with CpCyP23 was examined by the IF assay (IFA) in HCT‐8 cells. Cells were grown on glass coverslips placed in 24‐well plates. Upon reaching 70%–80% confluence, the cells were infected with HA‐tagged *C. parvum* oocysts. At 3 h postinfection (hpi), corresponding to the early phase of the parasite–host interaction, the biological specimens were immobilized using 4% paraformaldehyde and subsequently treated with 0.1% Triton X‐100 for membrane permeabilization. Following a blocking step, samples were probed with a cocktail of rabbit anti‐PHB2 and mouse anti‐HA primary antibodies (1:1000, Servicebio, GB151252‐100) during a 4°C overnight incubation phase. This was followed by a 60‐min association with CoraLitePlus555‐conjugated goat anti‐rabbit IgG (1:200, Proteintech, RGAR003), CoraLitePlus647‐conjugated goat anti‐mouse IgG (1:200, Proteintech, RGAM005), and fluorescein‐labeled *Vicia villosa* lectin (VVL) (Vector, Newark, CA, USA) for 1 h at room temperature in the dark. Nuclei were stained with DAPI (Beyotime). Images were acquired using an Olympus IX83 P2ZF super‐resolution microscope (Olympus). To better visualize colocalization, the display color of the CoraLitePlus647 channel was converted from magenta to cyan in the FV31S‐SW Viewer (Olympus), while the CoraLitePlus555 channel remained red. This adjustment modified only the pseudocolor display and did not alter the original pixel values.

### 2.11. Analysis of PHB2 Expression During *C. parvum* Infection

HCT‐8 cells were seeded into 12‐well plates and infected with *C. parvum* when they reached 70%–80% confluence, with uninfected cells serving as a control. Cell samples were collected at 3, 6, 12, 24, 36, 48, and 72 hpi. Total RNA and proteins were extracted to determine the mRNA and protein expression levels of PHB2 via quantitative real‐time PCR (qPCR) and Western blotting, respectively. All oligonucleotide sequences are provided in Supporting Information 2: Table S2.

### 2.12. Small Interfering RNA (siRNA) and Overexpression Vector

To modulate PHB2 levels, both knockdown and overexpression strategies were employed in HCT‐8 cells. For PHB2 knockdown, three specific siRNAs (siRNA‐1, siRNA‐2, and siRNA‐3) and a nontargeting control siRNA (control) were designed according to the human PHB2 mRNA sequence and synthesized by Sangon Biotech. For overexpression, the human PHB2 coding sequence was inserted into the pcDNA3.1(+) vector to construct the pcDNA3.1‐PHB2 plasmid, with the empty vector serving as the control. HCT‐8 monolayers were established in 6‐well manifolds and, upon reaching ~75% density, were subjected to Lipofectamine 3000‐mediated delivery of either siRNAs (at final concentrations of 20, 50, and 100 nM) or the aforementioned expression constructs. Following a 24‐h incubation period, the regulatory efficacy was scrutinized at both the mRNA and protein levels via qPCR and Western blotting. All oligonucleotide and siRNA sequences are provided in Supporting Information 2 and 3: Tables S2 and S3, respectively.

### 2.13. Effect of PHB2 on *C. parvum* Infection

Following transfection with the aforementioned siRNAs or plasmids, the cells were infected with *C. parvum* oocysts. Inactivated oocysts served as a negative control. The infection was performed at 37°C for 1 h, after which the noninvaded parasites were removed by three washes with PBS. Cell samples were collected at 3 and 18 hpi. Total RNA was isolated, and the parasite burden within host cells was then determined by quantifying *C. parvum* 18S rRNA levels via qPCR. The primer sequences used for amplifying human and *C. parvum* 18S rRNA are listed in Supporting Information 2: Table S2.

### 2.14. Statistical Analysis

Statistical analyses were conducted with GraphPad Prism 10 software (GraphPad, Boston, MA, USA). All quantitative values are documented as means ± SD derived from a minimum of three autonomous biological iterations. Details regarding the specific statistical methodologies employed, along with their respective probability values, are provided within the individual figure captions. For all comparisons, a threshold of *p* < 0.05 was established to define statistical significance.

## 3. Results

### 3.1. Identification of Host Proteins Interacting With CpCyP23

**Figure 1:**
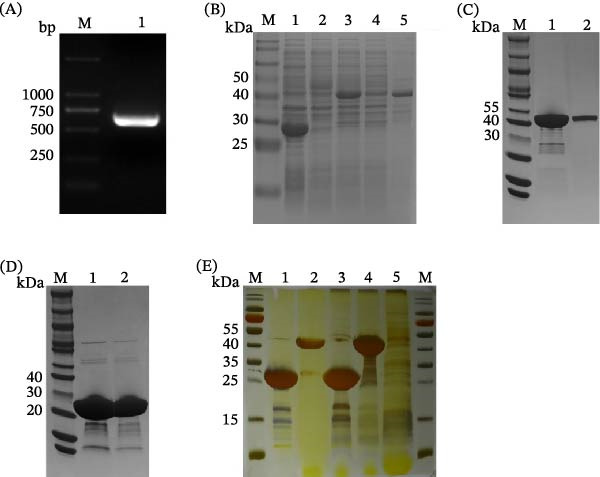
Expression of CpCyP23 in *Escherichia coli* and pull‐down assay. (A) PCR amplification of the *CpCyP23* genes of *C. parvum*. Lane M: 2000 bp DNA ladder maker. Lane 1: CpCyP23 product. (B) SDS‐PAGE analysis of CpCyP23 expressed in *E. coli*. Lane M: molecular weight markers. Lane 1: lysate from bacteria transformed with pGEX‐4T‐1 vector control. Lane 2: lysate from bacteria transformed with pGEX‐4T‐1‐CpCyP23 (without IPTG induction). Lane 3: lysate from bacteria transformed with pGEX4T‐1‐CpCyP23 (with IPTG induction). Lane 4: culture supernatant from Lane 3. Lane 5: culture pellet located in Lane 3. (C) SDS‐PAGE investigation of pure recombinant CpCyP23. Lane M: molecular weight markers. Lanes 1 and 2: CpCyP23 purified using glutathione‐agarose affinity chromatography. (D) SDS‐PAGE investigation of pure GST protein. Lane M: molecular weight markers. Lanes 1 and 2: GST purified using glutathione‐agarose affinity chromatography. (E) Silver staining analysis of pull‐down products. Lane M: molecular weight markers. Lane 1 and 2: purified GST and CpCyP23 protein. Lane 3 and 4: purified GST and CpCyP23 pull‐down products.

Host proteins interacting with CpCyP23 were identified using a pull‐down assay combined with MS analysis. Following the previously described method, purified GST‐CpCyP23 and GST‐tag control proteins (Figure 1A–C) were incubated with total protein lysates from HCT‐8 cells. The resulting pull‐down products were separated by SDS‐PAGE, and the interacting protein bands were visualized by silver staining (Figure 1D). The detailed identification results from MS analysis are provided in Supporting Information 1: Table S1. Bioinformatics analysis indicated that the identified interacting proteins were primarily localized to the cytoplasm and the nucleus. Given that these compartments are central hubs for the regulation of host cell functions, these proteins may play roles during pathogen infection by participating in the modulation of host cellular processes. It has been previously documented that *C. parvum* infection disrupts host cell functions. Therefore, based on its high MS identification score and its established close association with critical host cell functions.

### 3.2. Interaction Between CpCyP23 and PHB2

**Figure 2:**
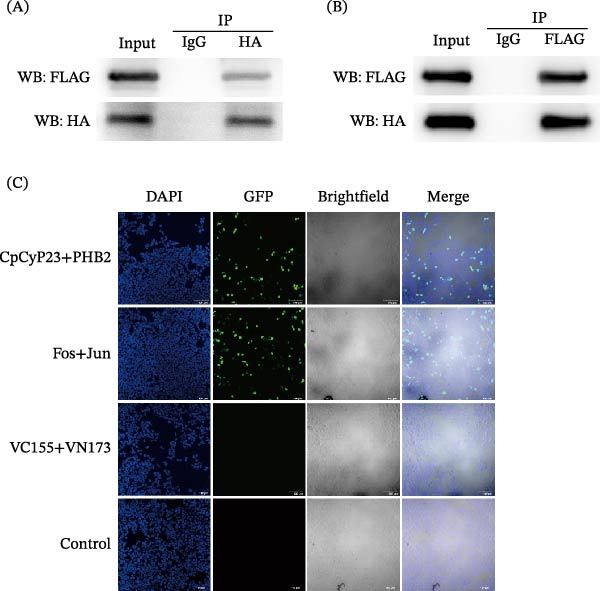
Validation of the interaction between CpCyP23 and the host protein PHB2. (A, B) Co‐IP analysis of the interaction between CpCyP23 and PHB2 in HEK293T cells. IP: immunoprecipitation. WB: western blot. Input: cell lysate before immunoprecipitation. IgG: control immunoprecipitation using mouse IgG. HA: epitope tag on CpCyP23. FLAG: epitope tag on PHB2. (C) BiFC visualizing the interaction between CpCyP23 and PHB2 in live mammalian cells. CpCyP23 and PHB2: experimental group (pBiFC‐VN173‐CpCyP23 + pBiFC‐VC155‐PHB2). Fos + Jun: positive control (a known interacting pair); VC155+VN173: negative control (mutants lacking interaction capacity). Control: untransfected HEK293T cells. Scale bar: 50 μm.

To validate the interaction between CpCyP23 and PHB2, a Co‐IP assay was performed. Plasmids expressing HA‐CpCyP23 and FLAG‐PHB2 were cotransfected into HEK293T cells. Cell lysates were subjected to immunoprecipitation using either an anti‐FLAG or an anti‐HA antibody, and the precipitates were subsequently analyzed by immunoblotting with anti‐HA and anti‐FLAG antibodies, respectively. The results demonstrated that PHB2 coprecipitated with CpCyP23 (Figure 2A,B).

To further confirm the interaction between CpCyP23 and PHB2 in live cells, a BiFC assay was conducted. HEK293T cells were transfected with plasmids for the experimental group (pBiFC‐VN173‐CpCyP23 and pBiFC‐VN155‐PHB2), the negative control group (pbJun‐VN173 and pbFos‐VC155, both carrying point mutations), and the positive control group (pbJun‐VN173 and pbFos‐VC155). Distinct punctate green fluorescent signals were observed in cells of both the experimental and positive control groups, whereas no fluorescence signal was detected in the negative control cells (Figure 2C). These results indicate that CpCyP23 and PHB2 interact within the cells.

### 3.3. Expression and Purification of His‐PHB2

**Figure 3:**
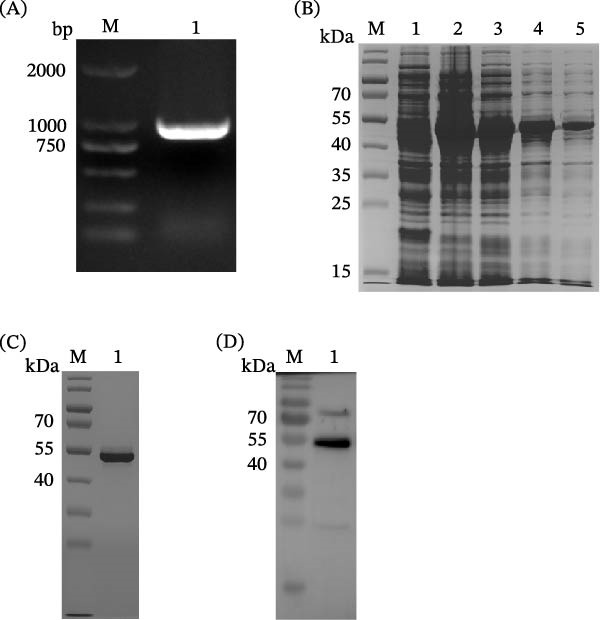
Expression of PHB2 in *Escherichia coli*. (A) PCR amplification of the *PHB2* gene of human. Lane M: DNA marker; Lane 1: PHB2 PCR product. (B) SDS‐PAGE analysis of PHB2 expression in *E. coli*. Lane M: molecular weight markers. Lane 1: lysate from bacteria transformed with the empty pET32a vector. Lane 2: lysate from bacteria transformed with pET32a‐PHB2 (uninduced). Lane 3: lysate from bacteria transformed with pET32a‐PHB2 (IPTG‐induced). Lane 4: supernatant from the culture in Lane 3. Lane 5: pellet from the culture in Lane 3. (C) SDS‐PAGE analysis of purified recombinant PHB2. Lane M: molecular weight markers. Lane 1: PHB2 purified using Ni‐TED agarose resin. (D) Western blot analysis of purified recombinant PHB2. Lane M: molecular weight markers. Lane 1: purified PHB2 detected using an anti‐His antibody.

The PHB2 gene was successfully amplified by PCR using specific primers, yielding a product of the expected size (929 bp; Figure 3A). SDS‐PAGE analysis indicated that the recombinant PHB2 protein was produced at the anticipated molecular weight (~52 kDa; Figure 3B). Recombinant CpCyP23 was purified via its His‐tag using imidazole elution (Figure 3C). Successful expression of the recombinant PHB2 was confirmed by western blot analysis (Figure 3D).

### 3.4. CpCyP23 Colocalizes With PHB2 in the Cytoplasm of HCT‐8 Cells

**Figure 4:**
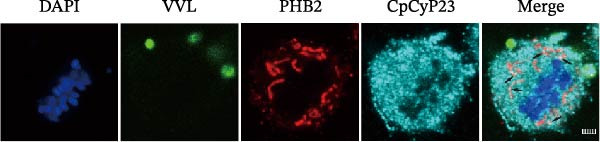
Immunofluorescence analysis of CpCyP23 and PHB2 localization. Scale bar: 2 μm.

To assess the subcellular localization relationship between CpCyP23 and PHB2, HCT‐8 cells were infected with HA‐tagged *C. parvum*. IF staining revealed that the clearest and most robust colocalization signal was observed at 6 hpi. At this time point, PHB2 (red) was predominantly localized in the cytoplasm, while CpCyP23 (cyan) was also distributed in the cytoplasm, and the parasitophorous vacuole (green) was localized at the periphery of the host cell. Marked colocalization of PHB2 and CpCyP23 was detected in the cytoplasm, with overlapping fluorescence signals appearing white (indicated by black arrows) (Figure 4).

### 3.5. The Expression Profile of Host PHB2 Upon *C. parvum* Infection

**Figure 5:**
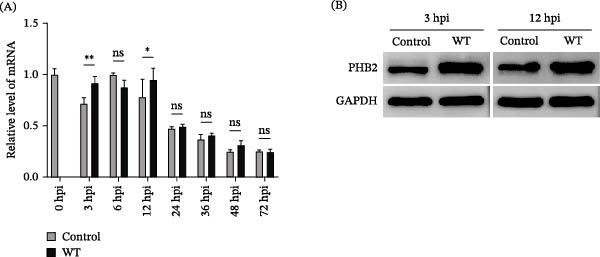
Effect of *C. parvum* infection on PHB2 expression in HCT‐8 cells. (A) qPCR analysis of PHB2 transcript levels in HCT‐8 cells at different time points after *C. parvum* infection. (B) Western blot analysis of changes in PHB2 protein expression levels at 3 h and 12 h postinfection.  ^∗^ *p* < 0.05 and  ^∗∗^ *p* < 0.01.

To examine the expression of PHB2 in HCT‐8 cells following *C. parvum* infection, the transcriptional levels of PHB2 were assessed at 3, 6, 12, 24, 36, 48, and 72 hpi. The relative mRNA expression of PHB2 across all time points was normalized using the 0‐h time point of the uninfected control group as the unified baseline. qPCR results showed that, compared with the uninfected control group, PHB2 was significantly upregulated at both 3 h (*p* < 0.01) and 12 h (*p* < 0.05) after *C. parvum* infection (Figure 5A). Subsequently, Western blot analysis was performed to detect PHB2 protein expression levels at 3 and 12 hpi. The results demonstrated a trend consistent with the qPCR data, namely, that *C. parvum* infection led to upregulated expression of PHB2 protein at both 3 and 12 hpi (Figure 5B).

### 3.6. si‐PHB2 Reduces *C. parvum* Infection

**Figure 6:**
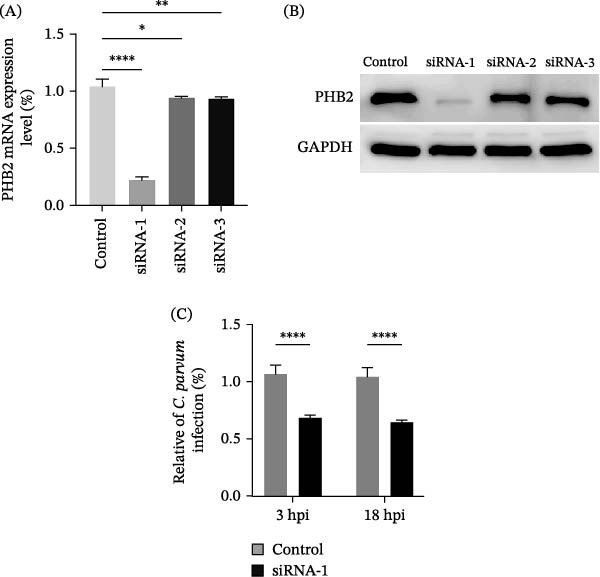
Assessment of siRNA knockdown efficiency and its inhibitory effect on *C. parvum* infection in HCT‐8 cells. (A, B) Detection of siRNA knockdown efficiency using qPCR and western blot. (C) Inhibition of *C. parvum* infection in HCT‐8 cells by siRNA. Human 18S rRNA served as the endogenous reference. Data are presented as mean ± SD (*n* = 3).  ^∗^ *p* < 0.05,  ^∗∗^ *p* < 0.01, and  ^∗∗∗∗^ *p* < 0.0001.

To examine the impact of PHB2 expression levels on *C. parvum* infection, siRNA targeting PHB2 was designed and transfected into HCT‐8 cells to silence its expression. The results showed that siRNA‐1 (targeting 5^′^‐GGATCCCTTGGTTCCAGTA‐3^′^) exhibited the most potent silencing efficiency. At a concentration of 100 nM, siRNA‐1 reduced PHB2 mRNA levels by ~70%–80% relative to the control, and Western blot analysis further confirmed a corresponding decrease in PHB2 protein expression (Figure 6A,B). Functional assays were subsequently conducted using the siRNA‐1. The parasite burden was quantified by qPCR at 3 and 18 hpi. The results revealed a significant reduction in the parasite burden in the PHB2‐silenced group compared to the control group at both 3 and 18 hpi (Figure 6C).

### 3.7. Overexpression of PHB2 Enhances *C. parvum* Infection

**Figure 7:**
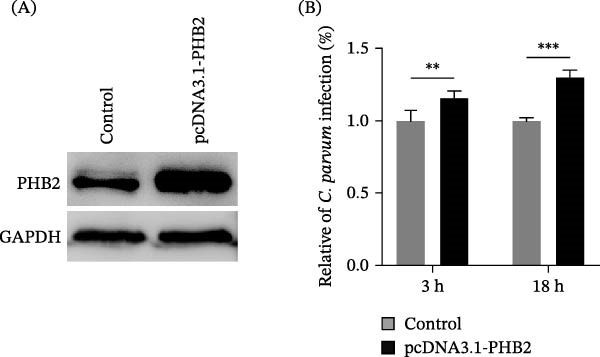
Promotion of *C. parvum* infection in HCT‐8 cells by PHB2 overexpression. (A) Enhanced expression of PHB2 protein in HCT‐8 cells by the overexpression vector. (B) Promotion of *C. parvum* infection in HCT‐8 cells by the overexpression vector. Human 18S rRNA served as the endogenous reference. Data are presented as mean ± SD.  ^∗∗^ *p* < 0.01 and  ^∗∗∗^ *p* < 0.001.

To further investigate the role of PHB2 during *C. parvum* infection, the PHB2 overexpression plasmid pcDNA3.1‐PHB2 was transfected into HCT‐8 cells (Figure 7A). qPCR analysis revealed that compared to the control group, the parasite burden was significantly increased in the PHB2‐overexpressing group at both 3 and 18 hpi (Figure 7B).

## 4. Discussion

The life cycle of *C. parvum* primarily includes stages such as sporozoite adhesion and invasion of host cells, followed by intracellular development and replication. The successful invasion of host cells by excysted sporozoites is a critical initial step in establishing infection [20]. Previous research has definitively localized CpCyP23 to extracellular vesicles released by sporozoites, and functional studies of this protein suggest its potential to interact with host cell proteins to facilitate sporozoite invasion [12, 21]. Therefore, the systematic identification of host proteins interacting with CpCyP23 will aid in elucidating the molecular mechanisms by which this parasite protein mediates the invasion.

Using a GST‐CpCyP23 fusion protein as a bait, we screened HCT‐8 cell lysates and identified 251 candidate proteins potentially interacting with CpCyP23. To minimize subjective bias during candidate prioritization, we applied a multistep selection framework integrating orthogonal criteria, including MS confidence, predicted subcellular localization, and functional pathway relevance. Among the proteins satisfying all criteria, PHB2 was prioritized for validation based on its strong peptide coverage and its known involvement in pathways relevant to *C. parvum* infection, including apoptosis and cell cycle regulation. Additionally, PHB2 has been reported to regulate processes like apoptosis and autophagy [22] and serves as a key host factor in infections by various pathogens. For instance, during *Salmonella* Typhimurium infection, PHB2 acts as a mitophagy receptor involved in bacterial survival and persistent infection [23]. In Enterovirus A71 infection, its structural protein VP1 interacts with PHB2 to induce autophagy and promote viral replication [24]. Additionally, the PHB1/2 complex has been suggested as a potential receptor for dengue virus entry into neural cells [25]. Notably, the molecular weight of PHB2 is ~33 kDa, which corresponds to the position of a specific pull‐down band observed in the silver‐stained gel for the GST‐CpCyP23 sample, further supporting PHB2 as a likely binding partner. Based on these lines of evidence, we preliminarily hypothesized that PHB2 plays an important role in *C. parvum* infection and selected it for further investigation.

The interaction between CpCyP23 and PHB2 was confirmed through Co‐IP and BiFC assays. Subsequently, IF analysis demonstrated their colocalization in the cytoplasm of HCT‐8 cells, corroborating their interaction.

Initially, we examined the PHB2 expression during the course of *C. parvum* infection. PHB2 transcript levels were significantly elevated at both 3 and 12 hpi, with corresponding increases observed at the protein level, suggesting that PHB2 is actively engaged in the infection process within HCT‐8 cells. To further delineate the functional role of PHB2 in *C. parvum* infection, we employed siRNA‐mediated knockdown to reduce PHB2 expression in HCT‐8 cells, with knockdown efficiency verified by qPCR and Western blot. The results showed a significant reduction in the parasite burden at both 3 and 18 hpi in PHB2‐knockdown cells compared to controls. Conversely, cells overexpressing PHB2 exhibited a significant increase in the parasite burden at the same time points. These findings indicate a positive correlation between PHB2 expression levels and *C. parvum* parasite load, suggesting that PHB2 is a key host factor promoting parasite invasion and intracellular survival. PHB2 has been shown to regulate apoptosis, potentially by modulating cytochrome c release and maintaining mitochondrial homeostasis, thereby exerting an antiapoptotic effect [22, 26]. In the early stages of *Cryptosporidium* infection, it is necessary to inhibit host cell apoptosis to prevent disruption of the parasitic niche, facilitating successful invasion and intracellular development [27, 28]. Therefore, PHB2 might promote *C. parvum* infection by suppressing infection‐associated host apoptotic pathways, thereby creating a stable cellular environment. It is plausible that CpCyP23, through its specific interaction with PHB2, forms a functional complex that potentiates the antiapoptotic activity of PHB2, further promoting cryptosporidial infection. However, these hypotheses require further experimental validation.

## 5. Conclusions

In this study, using purified CpCyP23 as bait, we employed GST pull‐down combined with MS to screen for interacting host proteins, identifying PHB2 from HCT‐8 cells as an interacting partner of CpCyP23. Functional investigations demonstrated that the expression level of the host protein PHB2 significantly influences the invasion and parasite burden of *C. parvum*, playing an important role in establishing infection.

## Author Contributions

**Mengfei Xu and Qi Feng**: writing – original draft, methodology, investigation, data curation. **Xichen Zhang**: writing – review and editing, formal analysis. **Qi Zhao**: investigation, formal analysis. **Qile Yu**: visualization, data curation. **Jianhua Li**: methodology, formal analysis. **Pengtao Gong**: methodology, investigation. **Xiaocen Wang**: investigation. **Xin Li**: writing – review and editing. **Xu Zhang**: writing – review and editing, resources. **Nan Zhang**: conceptualization, methodology, writing – review and editing, resources.

## Funding

This work was supported by the grants from the National Key Research and Development Program of China (Grant 2022YFD1800200), the Science and Technology Development Program of Jilin Province (Grant SKL202602017JC) and the National Natural Science Foundation of China (Grant 31972704).

## Ethics Statement

The experimental animals were conducted following the “Regulations for the Administration of Laboratory Animals” of the People’s Republic of China. All ethical regulations regarding animal use were followed. All experiments were approved by Jilin University (Approval ID SY202202104).

## Conflicts of Interest

The authors declare no conflicts of interest.

## Supporting Information

Additional supporting information can be found online in the Supporting Information section.

## Supporting information


**Supporting Information 1** Table S1: Mass spectrometry results of CpCyP23 pull‐down assay.

**Supporting Information 2** Table S2: Primer sequences used in this study..

**Supporting Information 3** Table S3: siRNA sequences used in this study.

## Data Availability

The data that support the findings of this study are available in the Supporting Information of this article.
